# Items

**Published:** 1903-04

**Authors:** 


### ITEMS.

THE Illinois Central Railroad will furnish to visitors to the American Medical Association a Pullman train from Louisville to New Orleans, under the auspices of Dr. Henry E. Tuley, secretary of the Mississippi Valley Medical Association. The fare will be $19.00—half rate—with $5.00 added for the sleeping berth. Reservations should be made at an early day, by addressing Dr. Tuley, 111 Kentucky St., Louisville, Ky.

---

BATTLE & COMPANY, of St. Louis, recently published Chart No. 11, of the series illustrating fractures of long bones, which depicts in a graphic manner fracture of the shaft of the radius. In this fracture the upper fragment is drawn forward by the biceps and rotated inward, over the ulna, by the pronator radii teres, to a position midway between pronation and supination. The lower fragment is drawn inward and slightly pronated by the pronator quadratus; at the same time the supinator longus, by elevating the styloid process, depresses the upper end of the lower fragment still more toward the ulna.

These phenomena are admirably illustrated in color by the drawing. This fracture is best reduced by flexing the forearm on the arm, placing the hand in a position midway between pronation and supination and making extension from the elbow; and the bone should be retained in this position by well-padded lateral splints extending from elbow to wrist, or by the application of a light plaster-cast to arm and forearm.

---

The Oppenheimer Institute, of New York, with branches in Philadelphia, Pittsburg and Detroit, has sent out an address to the medical profession, asking its support and confidence, and promising to conduct its affairs on scientific and ethical lines. These institutions (see advtg. p. xxxiv.) are established for the treatment of cases of alcoholic intemperance and drug addiction, including opium and its derivatives; cocaine, chloral, arsenic, tobacco, and, in certain cases, neurasthenia. For general information in reference to the business, printed matter, and the like, write or apply to the executive offices, 170 Broadway.

---

Monsieur A. Mariani, of Paris, who is known everywhere throughout the world as the originator of Vin Mariani, has published a second edition of the album of eminent physicians, which was first issued some years ago. It contains 32 wood cuts and biographical sketches of medical men in France, who are well known for their scientific attainments and high professional standing. The engravings are works of art and the album is attractive in its make up. For copies address Mariani & Company, 52 West 15th Street, New York.

---

The New York Pharmacal Association of Yonkers, has lately issued a brochure, entitled, *Clinical Examination of the Gastric Contents,* designed to furnish physicians with a condensed, but comprehensive working guide for the chemical examination of the contents of the stomach. It describes the most approved methods of making such examinations, deals with the interpretation of evidence found in abnormal conditions, and presents a brief diagnostic review of the commoner diseases of the stomach and their influence upon the gastric secretions. This method of advertising is to be commended, because it gives scientific information in an attractive and easily accessible form. The monograph is appropriately illustrated.

